# The Interaction of Histamine H_3_ and Dopamine D_1_ Receptors on Hyperkinetic Alterations in Animal Models of Parkinson’s Disease

**DOI:** 10.3390/ph17121726

**Published:** 2024-12-20

**Authors:** Alberto Avila-Luna, Antonio Verduzco-Mendoza, Adriana Olmos-Hernández, José Luis Cortes-Altamirano, Alfonso Alfaro-Rodríguez, José-Antonio Arias-Montaño, Antonio Bueno-Nava

**Affiliations:** 1 División de Neurociencias Básicas, Instituto Nacional de Rehabilitación Luis Guillermo Ibarra Ibarra, SSa, Calzada México-Xochimilco 289, Arenal de Guadalupe, Ciudad de México 14389, Mexico; 2 Bioterio y Cirugía Experimental, Instituto Nacional de Rehabilitación Luis Guillermo Ibarra Ibarra, SSa, Calzada México-Xochimilco 289, Arenal de Guadalupe, Ciudad de México 14389, Mexico; 3 Departamento de Quiropráctica, Universidad Estatal del Valle de Ecatepec, Ecatepec de Morelos 55210, Mexico; 4 Departamento de Fisiología, Biofísica y Neurociencias, Centro de Investigación y de Estudios Avanzados del Instituto Politécnico Nacional, Av. IPN 2508, Zacatenco, Ciudad de México 07360, Mexico

**Keywords:** dopamine, histamine, D_1_ receptor, H_3_ receptor, L-Dopa-induced dyskinesias, striatum, cerebral cortex, Parkinson’s disease, GABA, glutamate

## Abstract

Parkinson’s disease is associated with the loss of more than 40% of dopaminergic neurons in the substantia nigra pars compacta. One of the therapeutic options for restoring striatal dopamine levels is the administration of L-3,4-dihydroxyphenylalanine (L-Dopa). However, Parkinson’s disease patients on long-term L-Dopa therapy often experience motor complications, such as dyskinesias. L-Dopa-induced dyskinesias (LIDs) manifest as abnormal involuntary movements and are produced by elevated striatal dopamine levels, which lead to increased activity of the basal ganglia direct striato-nigral pathway. Dopamine D_1_ receptors are more than 95% confined to neurons of the direct pathway, where they colocalize with histamine H_3_ receptors. There is evidence of functional interactions between D_1_ and H_3_ receptors, and here we review the consequences of these interactions on LIDs.

## 1. Introduction

Parkinson’s disease (PD) is one of the most common neurodegenerative disorders worldwide, only second to Alzheimer’s disease. PD has a prevalence of more than 6 million people [1,2] and is originated by the progressive loss of the dopaminergic neurons located in the substantia nigra *pars compacta* (SNc) [1,3]. The administration of L-3,4-dihydroxyphenylalanine (L-Dopa) is one of the most effective treatments for PD motor symptoms [2,4]. However, in the long term, L-Dopa administration induces abnormal involuntary movements (AIMs), known as L-Dopa-induced dyskinesias (LIDs) [5].

**Figure 1:**
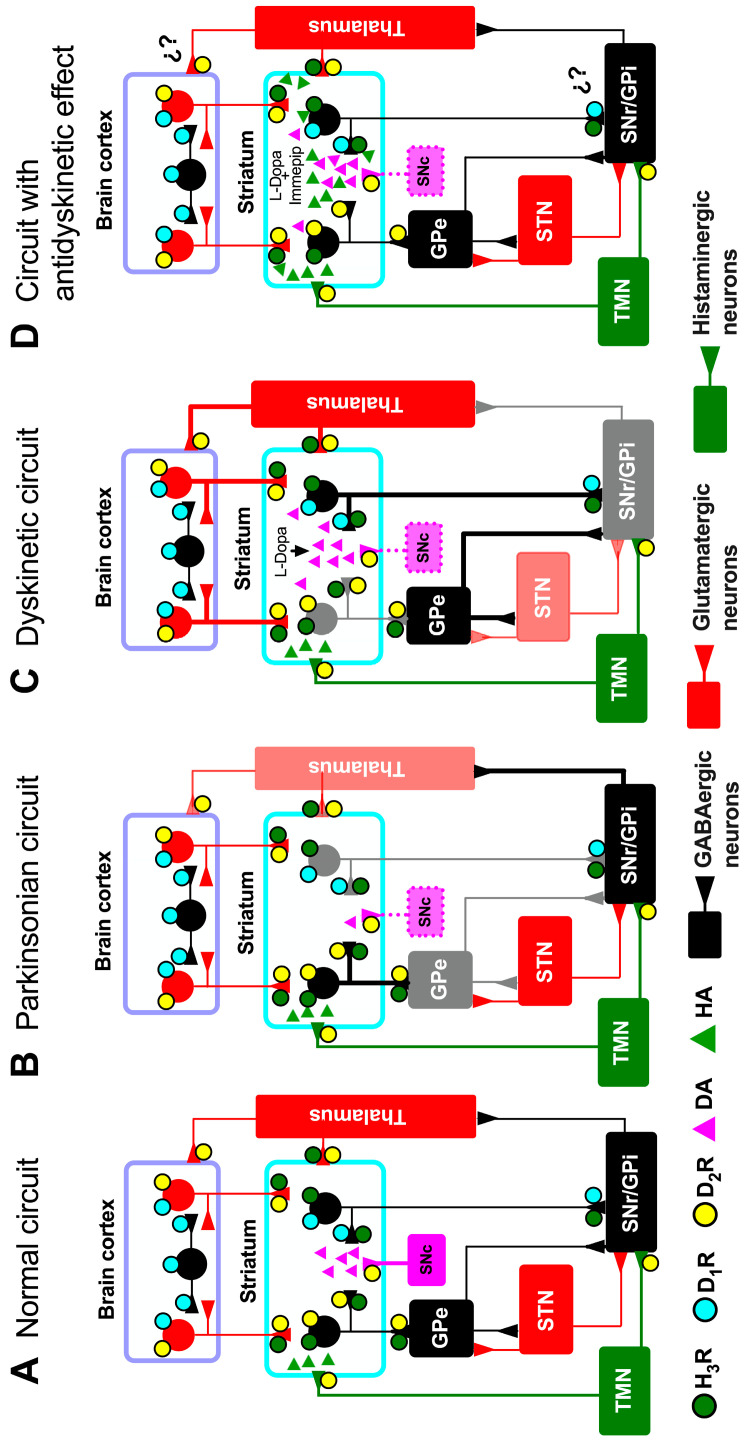
Schematic view of the basal ganglia synaptic circuitry. (**A**) Normal circuit; (**B**) parkinsonian circuit; (**C**) dyskinetic circuit, and (**D**) circuit with the antidyskinetic effect of the chronic administration of the H_3_R agonist immepip. Black lines represent inhibitory neuronal pathways, while red lines represent excitatory projections. The thickness of the lines and the color intensity or lightness indicate the degree of activation of each projection. The grey tone in the external segment of the globus pallidus (GPe; 1B), the substantia nigra *pars reticulata* (SNr; 1C), the internal segment of the globus pallidus (GPi; 1C), thalamus (1B), and the subthalamic nucleus (STN; 1C) reflect differences in activation compared to other regions shown in black. Dotted pink lines indicate dopaminergic depletion in the substantia nigra *pars compacta* (SNc). MSN axons project to the SNr and the GPi through a direct pathway and to the GPe, which in turn projects to the STN, forming the indirect pathway that projects to the SNr and GPi. The legends ‘L-Dopa’ and ‘L-Dopa + immepip’ for the striatum in panels C and D indicate the systemic administration of L-Dopa alone or in conjunction with the H_3_R agonist immepip, respectively. ¿?, unconfirmed effect. DA, dopamine; HA, histamine; TMN, tuberomammillary nucleus.

LIDs are associated with elevated dopamine (DA) levels in the nucleus striatum, which lead to molecular and cellular alterations in the function of the basal ganglia (BG) direct pathway (see below and Figure 1C) [4,6,7,8]. The post-synaptic changes include cellular redistribution of DA D_1_-like receptors (D_1_Rs), sensitization of D_1_R intracellular signaling, and abnormal gene expression in D_1_R-expressing neurons [4,9,10].

In LIDs, the hyperactivity of the direct BG pathway is critical for the observed hypoactivity of the GABAergic pallido/nigro-thalamic projections, resulting from increased activity of the glutamatergic thalamo-cortical pathway (Figure 1C) [11,12]. Amantadine is highly effective for treating LIDs by antagonizing glutamate receptors and reducing excitatory neurotransmission [2,13,14]. However, the withdrawal of amantadine aggravates LIDs after ~7 days, indicating action reversibility and a rebound effect [15].

The BG direct and indirect pathways are initiated by the projecting axons of the striatal GABAergic medium spiny neurons (MSNs), which constitute 95% of the striatal neuronal population [16]. The striatum is innervated by axons of dopaminergic and histaminergic neurons located in the SNc and the hypothalamus tuberomammillary nucleus, respectively [16]. Histamine H_3_ receptors (H_3_Rs) are co-expressed with D_1_Rs in the MSNs of the BG striato-nigral or direct pathway and with D_2_-like receptors (D_2_Rs) in striato-pallidal MSNs that originate from the first segment of the indirect pathway [17].

D_1_Rs comprise the D_1_ and D_5_ subtypes, both coupled to Gα_s_ proteins and thus to the activation of the cAMP/protein kinase A (PKA) signaling pathway. D_2_Rs include the D_2_, D_3_, and D_4_ subtypes, all coupled to Gα_i/o_ proteins. The D_2_R main signaling mechanisms include the inhibition of the cAMP/PKA pathway and reduction in calcium entry through voltage-activated Ca^2+^ channels involved in neurotransmitter release [18].

H_3_Rs couple to Gα_i/o_ proteins, and both the Gα_i/o_ subunits and the Gβγ dimers mediate G protein-dependent signaling. The actions reported are: (a) inhibition of cAMP formation; (b) inhibition of the Na^+^/H^+^ exchanger; (c) inhibition of N- and P/Q-type voltage-gated Ca^2+^ channels; (d) activation of G protein-gated inwardly rectifying K^+^ channels (GIRKs); (e) phospholipase C (PLC) activation; (f) activation of the mitogen-activated kinase (MAPK) pathway; (g) activation of the phosphatidylinositol 3-kinase (PI3K) pathway; and h) phospholipase A_2_ (PLA_2_) activation [19].

As indicated above, MSNs of the BG direct pathway co-express H_3_Rs and D_1_Rs [17], and in slices of rat substantia nigra *pars reticulata* (SNr) and striatum H_3_R activation inhibits D_1_R-stimulated cAMP accumulation and GABA release [20,21,22], indicating an opposite functional interaction. However, there is still limited information on whether this functional interaction affects LIDs, originating from molecular and cellular changes in the BG circuitry, with D_1_R overstimulation playing a causative role. In this review, we examine the reported effects of co-activating D_1_Rs and H_3_Rs on BG function and their potential influence on LIDs.

## 2. Striatal Synaptic Circuitry

The striatum is the main target of the BG synaptic afferents and receives two major glutamatergic inputs, the first from all areas of the cerebral cortex [23,24] and the second from the thalamus [25] (see Figure 1A). The number of synaptic contacts of cortical afferents with MSNs nearly equals that of the thalamic inputs [23,25]; however, in terms of synaptic interactions and functional properties, the cortico-striatal projections are highly dominant [25,26,27]. The striatum also receives glutamatergic inputs from other brain regions, namely the subthalamic nucleus (STN), the pedunculopontine nucleus, the amygdala, and the hippocampus [28,29,30,31,32].

The external segment of the external globus pallidus (GPe) is a GABAergic nucleus that receives massive synaptic inputs from the striatal MSNs and provides GABAergic projections to the STN and back to the striatum [16,33]. Other neurons in the striatal circuit include GABAergic and cholinergic interneurons, which represent almost 5% of all striatal neurons in rodents and up to 23% in primates [16].

The striatum thus integrates diverse synaptic inputs, predominantly glutamatergic information from the cerebral cortex and thalamus and dopaminergic input from the SNc. In turn, through the direct and indirect pathways, the striatum modulates the function of the BG output nuclei, namely SNr and the globus pallidus internal segment (GPi) in primates and humans or entopeduncular nucleus in rodents. In recent decades, it has been clear that histaminergic innervation (see Figure 1A) [16,34,35] also plays a relevant role in modulating the striatal function, thus contributing to the intricate regulatory mechanisms involved in motor control, cognition, and neuropsychiatric conditions [34,36,37,38].

## 3. Distribution of D_1_Rs and H_3_Rs in the Striatum

H_3_Rs are localized on histaminergic terminals as autoreceptors controlling histamine synthesis and release [19]. In the striatum, H_3_Rs are co-expressed by D_1_Rs in the MSNs of the direct BG pathway [17,39] and with D_2_ receptors (D_2_Rs) in MSNs of the indirect pathway [40,41]. In both MSN subpopulations, H_3_Rs are located in the somato-dendritic region and in axons that project to the GPe for the indirect pathway, and for the direct pathway on the soma and nerve terminals of MSNs that project to the output nuclei of the BG (SNr and GPi) [42]. They are also present in MSN collateral axons that innervate MSNs of the same or different subpopulations (see Figure 1A) [43].

In addition, H_3_Rs are located on striatal glutamatergic and dopaminergic afferents from the cerebral cortex and SNc, respectively, as well as in intrinsic interneurons [17]. For cholinergic interneurons, the expression of H_3_R mRNA was confirmed in both the ventral (nucleus accumbens) and dorsal striatum [44]. However, H_3_R activation hyperpolarizes the resting membrane potential and decreases the spontaneous firing rate of cholinergic interneurons in the nucleus accumbens but not in the striatum. Furthermore, the lack of effect of H_3_R activation on acetylcholine release from nucleus accumbens synaptosomes suggests a trans-synaptic mechanism involving reduced acetylcholine release, mediated by somatodendritic H_3_Rs, which leads to decreased activation of nicotinic receptors located on dopaminergic terminals [44].

Striatal interneurons also express dopamine receptors. Functionally, D_1_R activation depolarizes GABAergic low-threshold spike (LTS) interneurons and results in excitatory post-synaptic currents (EPSCs) in cholinergic and fast-spiking (FSI) GABAergic interneurons. D_2_R activation induces inhibitory post-synaptic currents (IPSCs) in cholinergic interneurons [45,46,47]. Thus, a functional interaction between H_3_Rs and D_1_Rs can also occur in striatal cholinergic and GABAergic interneurons.

Altogether, this information indicates that via H_3_R activation, histamine modulates key striatal synaptic pathways, influencing histaminergic, glutamatergic, and dopaminergic inputs to MSNs and striatal interneurons.

## 4. Functional Interaction Between H_3_Rs and D_1_Rs in the Striato-Nigral MSNs

There is information that supports the functional interaction between H_3_Rs and D_1_Rs in striato-nigral MSNs and, thus, in the direct BG pathway.

The first evidence for this interaction was the report that in slices of rat striatum and SNr, D_1_R activation facilitated depolarization-evoked GABA release, and H_3_R activation by the selective agonist immepip inhibited the component of release due to D_1_R stimulation [20,22]. Also in striatal slices, H_3_R activation inhibited D_1_R-induced cAMP accumulation, an action likely to take place in MSN bodies and their collaterals [21].

Further evaluation showed that the facilitatory action of D_1_Rs was mimicked by 8-bromo-cyclic AMP, prevented by PKA inhibition, and markedly reduced by ω-agatoxin TK, a blocker of P/Q-type voltage-gated Ca^2+^ channels, but not by ω-conotoxin MVIIA or nimodipine (blockers of N- or L-type Ca^2+^ channels, respectively). Moreover, the effect of 8-bromo-cyclic AMP was practically abolished by H_3_R activation [48]. Together, these data indicate that D_1_R-induced facilitation and H_3_R-mediated inhibition of GABA release from D_1_-MSN axon terminals converge at voltage-activated P-/Q-type Ca^2+^ channels present in the striato-nigral axons and the collaterals of D_1_R-expressing MSNs that remain in the striatum.

One heterodimeric configuration between D_1_Rs and H_3_Rs was confirmed on striato-nigral MSNs, where D_1_R activation facilitates the H_3_R-mediated stimulation of mitogen-activated protein kinases (MAPKs) in striatal slices [49,50]. This effect was not found in striato-pallidal MSNs [50]. Furthermore, H_3_Rs can form heteroreceptor complexes with D_1_Rs and glutamate N-methyl-D-aspartate (NMDA) receptors. In the D_1_R/H_3_R dimer, H_3_R activation reduces D_1_R affinity for selective agonists and shifts D_1_R coupling from Gα_s_ to Gα_i/o_ proteins [49,51], and in the D_1_R/H_3_R/NMDA receptor complex, H_3_R activation prevents D_1_R-induced ERK-1/2 phosphorylation [52].

In the same line, by using microdialysis, we reported an in vivo functional interaction between H_3_Rs and D_1_Rs, in which striatal DA efflux is reduced by the intra-striatal infusion of the D_1_R agonist SKF-38393, an effect counteracted by the co-infusion of the H_3_R agonist immepip [53]. This result indicates that the histaminergic system participates in a negative feedback mechanism that controls dopaminergic transmission in the striatum and involves post-synaptic D_1_Rs and D_2_Rs as well as pre-synaptic D_2_Rs.

In conclusion, H_3_Rs and D_1_Rs interact in striato-nigral MSNs, modulating GABA release, the cAMP/PKA signaling pathway, and voltage-activated Ca^2^⁺ channels. The H_3_R-D_1_R functional interaction also contributes to a feedback mechanism that controls striatal neurotransmission, and through heteroreceptor complexes, H_3_Rs and D_1_Rs regulate dopaminergic signaling in the striatum.

## 5. Functional Interaction Between H_3_Rs and D_1_Rs and Its Effect on Movement

It is well established that the chronic administration of L-Dopa in PD patients and animals lesioned with neurotoxins (6-OHDA or MPTP) will lead to dyskinesias [4,7,9]. A causal factor of LIDs is the development of D_1_R sensitization in the striatum, with receptors responding atypically to the administration of dopaminergic drugs [4]. The experimental evidence previously reviewed suggested a role for the functional interaction between H_3_Rs and D_1_Rs in the control of movement. In this line, the pioneer study by Papathanou et al. [54] showed that the systemic administration of a single dose of the H_3_R agonist immepip at 1, 5, or 10 mg/kg did not affect LIDs in rats lesioned with 6-hydroxydopamine (6-OHDA) in the SNc [53]. However, the study was not concluded for marmosets lesioned with 1-methyl-4-phenyl-1,2,3,6-tetrahydropyridine (MPTP) and primed with L-Dopa because the administration of immepip or imetit, also H_3_R agonist, resulted in adverse effects that lead to interrupting the experiments. Nevertheless, partial data from the study showed that both agonists reduced the antiparkinsonian response induced by L-Dopa, thus appearing to decrease LIDs [54].

**Figure 2:**
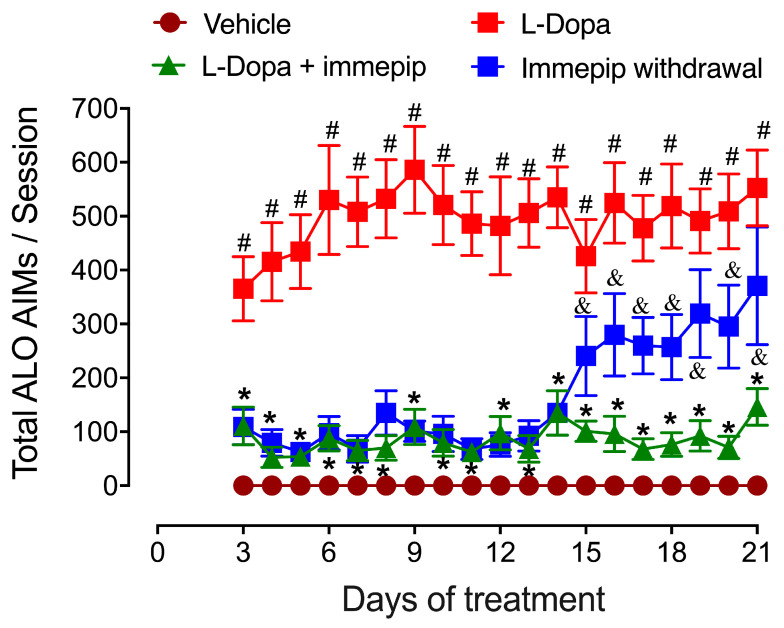
Effect of the systemic administration of the H_3_R agonist immepip (1 mg/kg, i.p.) on total ALO (axial, limb, and orolingual) abnormal involuntary movements (AIMs) induced by L-Dopa (6.25 mg/kg; benserazide 15 mg/kg, i.p.). ALO AIMs were individually counted and then summed per session. The statistical analysis of total AIMs was conducted with repeated-measures ANOVA followed by Bonferroni’s post hoc test (8 animals per group). &,# *p* < 0.01, compared to the vehicle group; * *p* < 0.001, compared to the L-Dopa group.

Our results showed that in 6-OHDA-lesioned rats, the chronic administration of the H_3_R agonist immepip in conjunction with L-Dopa reduced abnormal involuntary movement (AIM) scores and total AIMs for the three subtypes analyzed (axial, limb, and orolingual; see Figure 2) [37]. In contrast, the subacute and acute administration of a single dose (1 mg/kg, i.p.) of the H_3_R agonist did not affect LIDs, as previously reported by Papathanou et al. [54]. In both studies, at this dose, immepip did not produce any side effects [11,37]. Differences with the Papathanou et al. study [54] were the supplier of the H_3_R agonist and the administration route (subcutaneous in Papathanou et al. and intraperitoneal in our study).

Our results suggested thus that the H_3_R agonist helped prevent the development of LIDs but also that immepip is ineffective once dyskinesias are present [11]. This implication was supported by AIM reappearance after the withdrawal of the H_3_R agonist (see Figure 2) [11,37]. Also, the administration of the H_3_R agonist alone for 5 consecutive days prior to L-Dopa did not reduce AIMs [11], an effect possibly associated with H_3_R desensitization [55,56]. In this context, our data show that chronic H_3_R activation along with L-Dopa administration is necessary, in line with the information that a single dose of H_3_R agonists was not sufficient to inhibit LIDs in rats chronically treated with L-Dopa [54].

In summary, the evidence discussed indicates that H_3_R agonists modulate LIDs by reducing abnormal involuntary movements when chronically administered along L-Dopa. However, acute or subacute administration of H_3_R agonists is ineffective against established LIDs, likely due to H_3_R desensitization.

## 6. Functional Interaction Between H_3_Rs and D_1_Rs: A Potential Target to Reduce Dyskinesias

Together, the previously mentioned data raise the question of whether the functional interaction between H_3_Rs and D_1_Rs can be demonstrated in an altered striato-nigral circuit, such as in LIDs in PD.

As mentioned above, H_3_Rs and D_1_Rs couple to Gα_i/o_ and Gα_s_ proteins, respectively, and that H_3_R co-activation reduces D_1_R-induced GABA release and cAMP accumulation in striatal and SNr slices, indicating pre-synaptic and post-synaptic interactions taking place in the MSNs of the direct pathway [20,22,57].

In studies in which GABA release was analyzed in vivo by HPLC-coupled microdialysis in the rat striatum and total GABA content was determined post-mortem in striatal homogenates, our results showed that the systemic administration of L-Dopa to dyskinetic rats acutely increases GABA levels in striatal dialysates. The GABA/glutamate ratio is an indirect indicator of the fraction of glutamate converted to GABA before release into the synaptic cleft [58], and L-Dopa administration for 21 days augmented both GABA content and the GABA/glutamate ratio in the striatum ipsilateral to the lesioned SNc [11,37].

The reported changes in striatal GABAergic transmission suggest overactivity of the MSNs of the direct pathway, and in D_1_R knockout animals with 6-OHDA-induced nigro-striatal denervation, L-Dopa administration does not produce LIDs, ruling out the participation of the D_2_Rs in the genesis of the motor disorder [59,60]. Furthermore, L-Dopa administration for 21 days to parkinsonian animals increases glutamate and GABA levels in the ipsilateral and contralateral cerebral cortices [37]. Together, this information supports the theory that chronic L-Dopa administration to parkinsonian animals increases the activity of the cerebral cortex and striatal MSNs that express D_1_Rs and form the BG direct pathway.

In the striatum of parkinsonian rats treated with L-Dopa, the increase in both GABA and glutamate levels in dialysates was counteracted by the chronic co-administration of the H_3_R agonist immepip, but not by subchronic or acute administration [11]. For LIDs, the increase in striatal glutamate and GABA levels is consistent with the enhancement in glutamatergic and GABAergic transmission reported previously [61,62]. One likely explanation is the H_3_R-mediated inhibition of glutamate release from the cortico-striatal and thalamo-striatal terminals (see Figure 1D) [11,42,63], along with the decrease in striatal GABA content associated with the functional interaction between D_1_Rs and H_3_Rs [11,22].

**Figure 3:**
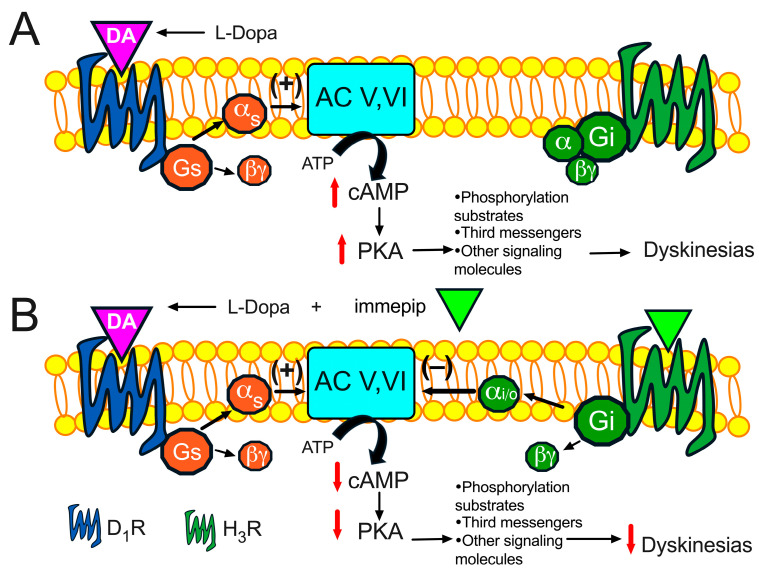
Schematic representation of L-Dopa-induced intracellular signaling involving the cAMP/protein kinase A (PKA) pathway (**A**). It is proposed that the co-administration of the H_3_R agonist immepip counteracts the effects of L-Dopa and reduces dyskinesias (**B**).

H_3_R activation leads to the inhibition of adenylyl cyclases (ACs) and the subsequent reduction in cAMP formation (see Figure 3) [42], and H_3_R activation inhibits D_1_R-induced cAMP accumulation in striatal slices [21]. L-Dopa administration leads to the enhanced activation of the cAMP/PKA pathway, and in MSNs expressing D_1_Rs, PKA phosphorylates several substrates, including DARPP-32 (DA- and cAMP-regulated phosphoprotein of 32 kDa), extracellular signal-regulated kinases (ERK-1/2), and proteins of the mTORC1 signaling pathway [4,64,65,66]. These molecular changes have been observed in MSNs of the striato-nigral direct pathway but not in MSNs of the indirect pathway [65]. In line with this, LIDs are reduced when L-Dopa administration is combined with drugs such as the PKA inhibitor Rp-cAMPS [66] or SL327, an ERK phosphorylation inhibitor [63,64]. Therefore, it is likely that the functional interaction between D_1_R and H_3_R explains the reduction in LIDs. However, molecular studies to corroborate this hypothesis are lacking.

From the reviewed information, it can be inferred that the functional interaction between H_3_Rs and D_1_Rs modulates LIDs by reducing L-Dopa-induced increases in striatal GABA and glutamate levels through H_3_R-mediated inhibition of cAMP/PKA signaling. These findings highlight the potential role of H_3_R activation to counteract the overactivity of the striato-nigral-cortical circuit observed in PD, though molecular studies are required to further explore this mechanism.

## 7. Interaction Between H_3_Rs and D_2_Rs in the Striato-Pallidal MSNs

H_3_Rs and D_2_Rs are co-expressed by striato-pallidal MSNs, and a few studies suggest a functional interaction between H_3_Rs and D_2_Rs in the striatum [67,68].

H_3_Rs and D_2_Rs couple to Gα_i/o_ proteins, and an additive action on [^35^S]-GTPγS binding to rat striatal membranes was observed following the co-activation of both receptors, a result that can be explained by only a fraction of the Gα_i/o_ protein pool being activated by each of the two receptors or by the activation by each receptor of different Gα_i/o_ subunit subtypes (α_i1_, α_i2_, α_i3_, α_oA_, or α_oB_) [67].

In reserpinized mice, H_3_R activation antagonizes locomotor behavior induced by D_2_R stimulation [68,69]. It was also reported that H_3_R activation reduces D_2_R affinity for the agonist quinpirole in sheep brain membranes and that the human H_3_R and D_2_R form a heterodimer as evidenced by BRET assays in transfected HEK-293 cells [69]. H_3_Rs co-immunoprecipitate with D_2_Rs in rat striatal lysates, and the proximity ligation assay (PLA) in mouse striatal sections supports the formation of an H_3_R/D_2_R complex. Furthermore, H_3_R co-activation modulated D_2_R-mediated Akt–GSK3β signaling in mouse D_2_R-expressing MSNs as visualized by immunohistochemistry and Western blot [68].

Together, this information indicates that H_3_Rs and D_2_Rs interact physically and functionally to modulate the activity of striato-pallidal neurons. However, as mentioned above, in D_1_R-knockout animals with 6-OHDA-induced lesions to striato-nigral neurons, the administration of L-Dopa does not result in dyskinesias, discarding a role for D_2_R in the pathogenesis of the disorder [57,58].

## 8. H_3_R/D_1_R Interaction in Huntington’s Disease (HD)

HD is a motor progressive neurodegenerative disorder caused by the expansion of a cytosine–adenine–guanine (CAG) trinucleotide repeat, coding a polyglutamine repeat within the N-terminal region of the protein huntingtin [70]. Like LIDs, dyskinesias in HD involve overactivity of the thalamo-cortical circuit. Dysfunction and death of striatal MSNs is a key HD neuropathological hallmark, and alterations in the striatal dopaminergic system may contribute to HD pathophysiology.

D_1_R over-activation occurs in the initial stages of HD, leading to abnormal dopaminergic neurotransmission and cell death [71]. In the study by Moreno-Delgado et al. [72], D_1_R/H_3_R heteromers were detected in immortalized striatal cells expressing mutant STHdhQ111 huntingtin, and D_1_R-induced ERK-1/2 phosphorylation, increase in intracellular Ca^2+^ levels and cell death were prevented by the H_3_R antagonist thioperamide via cross-antagonism. In the mutant HdhQ7/Q111 mouse, an HD model, thioperamide reduced motor and memory deficits. Furthermore, D_1_R/H_3_R heteromers were detected in the striatum (caudate-putamen) from control individuals and low-grade (0, 1, and 2 grades) HD patients but not in samples from high-grade (grades 3 or 4) patients, indicating that D_1_R/H_3_R heteromers are lost at late HD stages.

These data indicate that D_1_R/H_3_R heteromers participate in HD pathophysiology and may thus represent novel targets for the treatment of the disease.

## 9. Conclusions

The functional interaction between D_1_Rs and H_3_Rs most likely explains the H_3_R-induced reduction of LIDs in parkinsonism models, and may be the basis for novel pharmacological approaches to preventing or treating LIDs in PD patients. However, molecular and pharmacological studies are still required to elucidate in detail the signaling pathways involved in the D_1_R/H_3_R interaction. These findings will contribute to the understanding of the role of D_1_Rs and H_3_Rs in BG dysfunction occurring in hyperkinetic disorders and involving alterations of the dopaminergic system.

## Data Availability

Not applicable.

## Abbreviations

6-OHDA	6-Hydroxy-dopamine
AC	Adenylyl cyclase
AIMs	Abnormal involuntary movements
BG	Basal ganglia
D_1_Rs	Dopamine D_1_ receptors
D_2_Rs	Dopamine D_2_ receptors
DA	Dopamine
DARPP-32	DA- and cAMP-regulated phosphoprotein of 32 kDa
ERK	Extracellular signal-regulated kinase
GPe	External segment of the globus pallidus
GPi	Internal segment of the globus pallidus
H_3_Rs	Histamine H_3_ receptors
L-Dopa	L-3,4-dihydroxyphenylalanine
LIDs	L-Dopa-induced dyskinesias
MAPKs	Mitogen-activated protein kinases
MPTP	1-methyl-4-phenyl-1,2,3,6-tetrahydropyridine
MSNs	Medium spiny neurons
PD	Parkinson’s disease
PKA	Protein kinase A
SNc	Substantia nigra *pars compacta*
SNr	Substantia nigra *pars reticulata*
STN	Subthalamic nucleus
